# Case Studies in Infectious Disease

**DOI:** 10.3201/eid1601.091254

**Published:** 2010-01

**Authors:** Philip S. Brachman

**Affiliations:** Atlanta ID Group, Atlanta, Georgia, USA; Emory University School of Medicine, Atlanta; Mercer University, Atlanta

**Keywords:** Infectious disease, case studies, bacteria, viruses, book review

The authors have assembled a collection of case studies about the 40 infectious diseases that cause the most illness and death worldwide. Each chapter begins with a brief case presentation. This example is followed by a section on microbiologic aspects of the organism, including the pathophysiology of infection. The host response is then described, followed by a discussion of clinical manifestations, diagnostic methods, and treatment options, including prevention. A summary highlights salient points of each section. References, suggestions for further reading, and websites for additional information are all provided. Chapters conclude with a series of questions (answers are given at the end of the book).

The book is meant for use by medical students in a microbiology course, but it can also be used by any clinician who wants a concise review of the pathogens that cause infectious diseases. The case presentations are short and not presented as conditions having an unknown cause, but they rather serve as a clinical starting point to open discussion. The microbiology sections are geared more toward the student in a microbiology course and tend to have more details than are needed by a practicing clinician. The sections on patient symptoms are generally quite good and are inclusive. The varied clinical manifestations, particularly of the tropical diseases, are presented in an easy-to-understand format. The level of detail given provides a thorough yet succinct picture of each disease. The sections on diagnosis are generally inclusive, although a few did not mention some available diagnostic options used in the United States; this may have been due to differences in the availability of some tests in the United Kingdom, where many of the authors are based. The treatment sections tend to be abbreviated and frequently do not include the length of therapy and some other details that a practicing clinician would want to know. For those needing specific therapy guidelines, another source will be necessary.

The summary sections are quite good and are an excellent quick reference source if one wants just the highlights and a brief summary about the pathogen and disease. The questions at the end tend to be multiple choice with several possible correct answers for each one; they are not structured to prepare for testing purposes (such as for a board review). The websites are helpful sources for downloadable slides as well as for further information if more details are wanted.

The only chapter that was confusing was that on coxsackie viruses. The authors kept referring to other enteroviruses. The chapter could benefit from either fewer references to other enteroviruses or renaming it to be a section on enteroviruses in general.

Case Studies in Infectious Diseases is a valuable compilation of information on the most common diseases that cause illness and death worldwide. The presentation format with distinct sections makes it readable and well suited for either students just learning about the pathogens causing infectious disease or clinicians who need an update. The level of detail is well thought out and gives the reader a useful summary of each pathogen and disease state. The condensed presentations make it a good reference source for those with insufficient time to read through more detailed textbooks.

